# Transceive phase corrected 2D contrast source inversion‐electrical properties tomography

**DOI:** 10.1002/mrm.28619

**Published:** 2020-12-06

**Authors:** Peter R. S. Stijnman, Stefano Mandija, Patrick S. Fuchs, Cornelis A. T. van den Berg, Rob F. Remis

**Affiliations:** ^1^ Computational Imaging Group for MRI Diagnostics and Therapy Centre for Image Sciences UMC Utrecht Utrecht The Netherlands; ^2^ Department of Biomedical Engineering Eindhoven University of Technology Eindhoven The Netherlands; ^3^ Circuit & Systems Group of the Electrical Engineering Delft University of Technology Delft The Netherlands

**Keywords:** contrast source inversion, dielectric tissue mapping, electrical properties tomography, EPT, MRI, RF‐shield, transceive phase

## Abstract

**Purpose:**

To remove the necessity of the tranceive phase assumption for CSI‐EPT and show electrical properties maps reconstructed from measured data obtained using a standard 3T birdcage body coil setup.

**Methods:**

The existing CSI‐EPT algorithm is reformulated to use the transceive phase rather than relying on the transceive phase assumption. Furthermore, the radio frequency (RF)‐shield is numerically implemented to accurately model the RF fields inside the MRI scanner. We verify that the reformulated two‐dimensional (2D) CSI‐EPT algorithm can reconstruct electrical properties maps given 2D electromagnetic simulations. Afterward, the algorithm is tested with three‐dimensional (3D) FDTD simulations to investigate if the 2D CSI‐EPT can retrieve the electrical properties for 3D RF fields. Finally, an MR experiment at 3T with a phantom is performed.

**Results:**

From the results of the 2D simulations, it is seen that CSI‐EPT can reconstruct the electrical properties using MRI accessible quantities. For 3D simulations, it is observed that the electrical properties are underestimated, nonetheless, CSI‐EPT has a lower standard deviation than the standard Helmholtz based methods. Finally, the first CSI‐EPT reconstructions based on measured data are presented showing comparable accuracy and precision to reconstructions based on simulated data, and demonstrating the feasibility of CSI‐EPT.

**Conclusions:**

The CSI‐EPT algorithm was rewritten to use MRI accessible quantities. This allows for CSI‐EPT to fully exploit the benefits of the higher static magnetic field strengths with a standard quadrature birdcage coil setup.

## 1 INTRODUCTION

Electrical properties tomography (EPT) is an MR‐based technique aiming at measuring the electrical properties (conductivity and permittivity) of tissues. This is achieved in a non‐invasive manner through MRI‐based mapping of the circularly polarized magnetic component ($B_{1}^{+}$, the transmit efficiency) of the transmit radio frequency (RF) field. The acquired conductivity and permittivity can be used as a contrast mechanism, especially the conductivity of tissue has been shown to have potential as a biomarker in oncology^1^, ^2^, ^3^, ^4^, ^5^, ^6^ and stroke imaging.^7^, ^8^ Furthermore, the conductivity and permittivity are important in the field of MR safety, where they are used to compute the specific absorption rate (SAR). The SAR defines the amount of deposited energy during MRI exams and relates directly to the heating of the tissue under examination.^9^, ^10^, ^11^, ^12^

There is a variety of different EPT approaches that have been recently published as shown in the review work.^1^ A large group of these approaches are derivative based and stem from the Helmholtz equation for magnetic fields.^13^, ^14^, ^15^, ^16^, ^17^ In these approaches, a second‐order derivative using finite difference kernels needs to be computed on the measured $B_{1}^{+}$ fields. This leads to noise amplification in the reconstructed EPT maps and introduces errors in the reconstruction of the electrical properties most notably at tissue boundaries.^18^, ^19^

Next to Helmholtz (derivative‐based approaches), gradient‐EPT^20^ and cr‐MREPT,^21^ there are also approaches based on the integral formulation of the Maxwell equations^22^, ^23^ where the objective is to minimize a cost function by iteratively updating the electrical properties. Among these integral‐based methods, the contrast source inversion (CSI) method^24^ has been shown to be more noise robust than derivative‐based approaches,^25^ and allows better EPs reconstructions at tissue boundaries. However, these benefits come at the expense of a higher computational cost and, generally, integral‐based optimization methods are more difficult to implement.

Furthermore, CSI‐EPT reconstructions have two major limitations. First, CSI‐EPT requires knowledge of the incident RF electric and magnetic fields. These are the RF fields of the empty transmit coil, that is, when an object/patient is not present. These RF fields can only be obtained through electromagnetic simulations and will vary from coil to coil. Second, similar to the derivative‐based MR‐EPT approaches, the CSI‐EPT reconstruction algorithm is formulated in terms of the complex $B_{1}^{+}$ field. While the magnitude of the $B_{1}^{+}$ field is measurable with MRI, the phase is not accessible. To overcome this limitation, the measurable transceive phase is used. From this, the transmit phase is derived as half of the transceive phase, which is known as the transceive phase assumption (TPA). However, this assumption is only valid at low field strengths and for symmetrical objects, where the polarization remains circular. For more complex structures, nonsymmetrical objects, or multiple tissue interfaces, these conditions are not met, and the assumption is not valid.^15^ This occurs especially at higher static magnetic field strengths due to the larger magnitudes of scattered RF fields, leading to elliptical polarization.

To reconstruct the tissue parameters, essentially two options can be followed. The first option is to discard the phase information of the $B_{1}^{+}$ and formulate the CSI‐EPT algorithm in terms of only the magnitude. This has been done in Ref. [26] and has been implemented for MR‐EPT in Ref. [27]. While this approach showed its potential in two‐dimensional (2D) simulation settings, it comes with an intrinsic drawback. It is well known that the conductivity information is mostly imprinted in the phase of the $B_{1}^{+}$,^28^ therefore discarding this information from the reconstruction algorithm makes the reconstruction problem even more challenging.

Therefore, in this work, we reformulate the CSI‐EPT algorithm to use the $B_{1}^{+}$ magnitude and the transceive instead of the transmit phase, both measurable with MRI, without relying on the transceive phase assumption, which was until now a cornerstone of MR‐EPT reconstruction methods. Furthermore, CSI‐EPT reconstructions require knowledge of the background RF‐fields. These are obtained from electromagnetic simulations which require inclusion of the RF‐shield. We will show that supplying the CSI‐EPT algorithm with the correct incident fields is necessary to obtain the correct electromagnetic properties. These RF‐shields are especially important for reconstructions from realistic MRI measurements, as these fields are only accessible via simulations. Thus, we numerically incorporate this RF‐shield to mimic the realistic MRI scenario. Using these proposed changes, we will present the first reconstructions of the electrical properties of a phantom with MRI acquired data using CSI‐EPT.

## 2 THEORY

### 2.1 Transceive phase correction

In this section, we reformulate the CSI‐EPT algorithm as described in Ref. [22]. We start by defining the contrast source as

(1) $$w\left(\rho \right)=\chi \left(\rho \right)E_{z}\left(\rho \right),$$

where $E_{z}$ is the *z*‐component of the total electric field and *χ* is the contrast with respect to free space which is defined as

(2) $$\chi \left(\rho \right)=\varepsilon_{r}\left(\rho \right)‐1‐\frac{\sigma (\rho )}{j\omega \varepsilon_{0}},$$

with ***ρ*** as the position vector, $\varepsilon_{0}$ and $\varepsilon_{r}$ as the permittivity in vacuum and relative permittivity, respectively, *σ* is the conductivity and *ω* is the angular Larmor frequency. Reconstructing the contrast is the goal of this method since the electrical properties can directly be calculated once the contrast is known. The contrast and contrast source are the two parameters that are iteratively updated in CSI‐EPT. This is realized by minimizing the cost functional presented in Ref. [22] given by

(3) $$F\left(w_{l},\chi \right)=\eta_{S}\sum_{l}\|B_{1,l}^{+}‐\left(B_{1,l}^{+,\mathrm{inc}}+G_{S}^{+}\left\{w_{l}\right\}\right){\|}_{S}^{2}+\eta_{D}\sum_{l}{\left\|\chi \left(E_{z,l}^{\mathrm{inc}}+G_{D}\left\{w_{l}\right\}\right)‐w_{l}\right\|}_{D}^{2},$$

where *l* ≥ 1 indicates a summation over various transmit channels including linear combinations such as a standard quadrature drive, $\eta_{S,D}$ are normalization factors for the data functional (first term on the right‐hand side of (3)) and object functional (second term on the right‐hand side of (3)). It should be noted that in MRI the domain where the object is located, *D*, is the same as the location where the data are collected, *S*. Furthermore, $G_{S}^{+}$ and $G_{D}$ are integral operators that map the contrast source to the scattered $B_{1}^{+}$ field, $B_{1}^{+,sc}$, and the scattered electric field, $E_{z}^{\mathrm{sc}}$, respectively. These integral operators are given by

(4) $$E_{z}^{\mathrm{sc}}\left(\rho \right)=G_{D}\left\{w\left(\rho^{\prime }\right)\right\}=k_{0}^{2}\int_{\rho \in D}\hat{G}\left(\rho ‐\rho^{\prime }\right)w\left(\rho^{\prime }\right)dV,$$

(4) $$B_{1}^{+,\mathrm{sc}}\left(\rho \right)=G_{S}^{+}\left\{w\left(\rho^{\prime }\right)\right\}=\frac{\omega }{2c_{0}^{2}}\left(\partial_{x}+j\partial_{y}\right)\int_{\rho \in D}\hat{G}\left(\rho ‐\rho^{\prime }\right)w\left(\rho^{\prime }\right)dV.$$

Here, $k_{0}$ and $c_{0}$ are the wavenumber and the speed of light in vacuum, respectively. The spatial derivatives with respect to *x* and *y* are indicated with $\partial_{x,y}$. The source locations are defined by ***ρ***′ and $\hat{G}\left(\rho ‐\rho^{\prime }\right)$ is defined as the 2D free‐space Green’s function, which is given by

(5) $$\hat{G}\left(\rho ‐\rho^{\prime }\right)=‐\frac{j}{4}H_{0}^{\left(2\right)}(k_{0}|\left(\rho ‐\rho^{\prime }\right)\left|\right),$$

where $H_{0}^{\left(2\right)}$ is the zeroth‐order Hankel function of the second kind.

The problem with the cost functional given by (3) is that the phase of the $B_{1}^{+}$ field is not directly accessible through measurements. What can be measured is the so‐called transceive phase $\phi_{\pm }$. This transceive phase consists of the transmit phase $\phi_{+}$ and receive phase $\phi_{‐}$ according to

(6) $$\phi_{\pm }=\phi_{+}+\phi_{‐}.$$

Therefore, we can write the $B_{1}^{+}$ field in terms of the transceive phase as

(7) $$B_{1}^{+}=|B_{1}^{+}|e^{j\phi_{+}}=\left|B_{1}^{+}\right|e^{j\phi_{\pm }}e^{‐j\phi_{‐}}.$$

Substituting (7) into (3) results in a new cost functional given by

(8) $$F\left(w_{l},\chi ,\phi_{‐}\right)=\eta_{S}\sum_{l}\left\|\right|B_{1,l}^{+}|e^{j\phi_{\pm ,l}}e^{‐j\phi_{‐,l}}‐\left(B_{1,l}^{+,\mathrm{inc}}+G_{S}^{+}\left\{w_{l}\right\}\right){\|}_{S}^{2}+\eta_{D}\sum_{l}{\left\|\chi \left(E_{z,l}^{\mathrm{inc}}+G_{D}\left\{w_{l}\right\}\right)‐w_{l}\right\|}_{D}^{2}.$$

The transmit phase is now written in terms of the transceive (known) and receive phase (unknown). Iterating through the algorithm now proceeds similarly as in standard CSI‐EPT, except that the measurable transceive phase is used and that the receive phase is iteratively updated using the estimates of the contrast function and electric field strength at the current iteration, *n*.

In this section, the electric and magnetic RF fields are the fields encountered in the receive state of the birdcage coil. During transmission, the birdcage coil is fed in quadrature mode (ie, a $90^{\circ }$ phase difference between the two ports) and this creates a circularly polarized $B_{1}$ field that is efficient in tipping the spins. In receive mode, the birdcage coil is switched to a receive state which means the $90^{\circ }$ phase difference becomes a $‐90^{\circ }$ phase difference. This results in a counter‐rotating circularly polarized field that is efficient in receiving the signal. This receiving state of the birdcage coil is called reverse quadrature or anti‐quadrature. This mode of the transmit coil creates different RF fields. The incident RF fields in reverse quadrature display a simple phase shift for a birdcage coil setup compared with forward quadrature, but the scattered fields are inherently different which is why the TPA is not valid when these scattered fields are comparable in magnitude to the incident field.

To compute the scattered receive field at each iteration, we use

(9) $$B_{1,n}^{‐}\;\left(\rho \right)\;=\;G_{S}^{‐}\;\left\{\chi_{n‐1}\;\left(\rho^{\prime }\right)\;E_{z,n}\;\left(\rho^{\prime }\right)\;\right\}\;=\;‐\frac{\omega }{2c_{0}^{2}}\;\left(\partial_{x}\;‐\;j\partial_{y}\right)\;\int_{\rho \in }D\;\hat{G}\;\left(\rho \;‐\;\rho^{\prime }\right)\;\chi_{n‐1}\;\left(\rho^{\prime }\right)\;E_{z,n}\;\left(\rho^{\prime }\right)\;dV,$$

where $B_{1,n}^{‐}$ defines the complex conjugate of the scattered receive field and $E_{z,n}$, the electric field during reception (anti‐quadrature setting) at iteration *n*, is defined as

(10) $$E_{z,n}\left(\rho \right)=G_{D}\left\{\chi_{n‐1}\left(\rho \right)E_{z,n‐1}\left(\rho \right)\right\}+E_{z}^{\mathrm{inc}}\left(\rho \right).$$

Here $E_{z}^{\mathrm{inc}}$, the incident electric field during reception, is modeled together with $B_{1}^{‐,\mathrm{inc}}$ for an empty coil, that is, in reverse quadrature mode for a birdcage coil. Since the incident fields for the transmit state are already modeled for each port, the incident receive fields are acquired by driving these ports in reverse quadrature.

By adding the receive phase as an extra unknown into the minimization problem, the minimization becomes more difficult and the computation time is increased since the number of required integral operations is increased to obtain the receive phase. However, the TPA is no longer required to reconstruct the contrast from the $B_{1}^{+}$ data. The new pseudo code is shown below with a dagger (†) indicating the quantities in the receive state of the birdcage coil. Furthermore, the Polak‐Ribière update directions can be found in Refs. [29, 30] and [22].

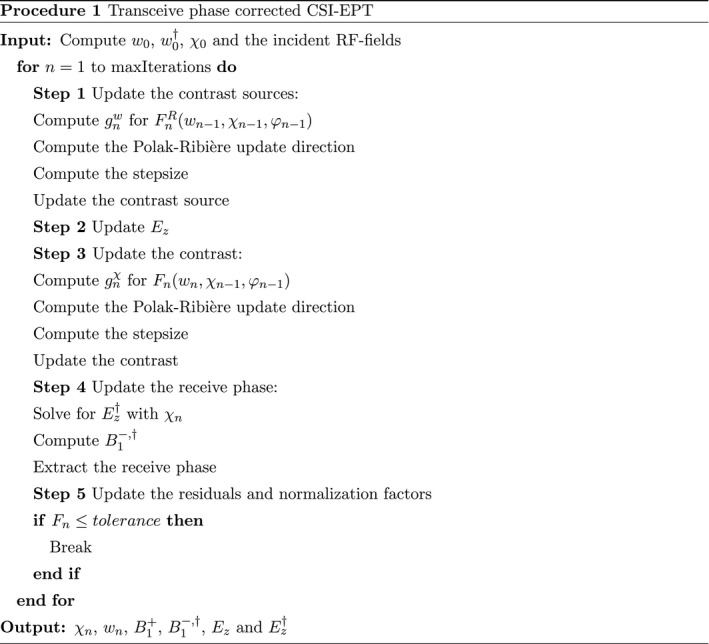

### 2.2 Numerical implementation of the RF‐shield

Inside every MRI system, there is an RF‐shield present. The purpose of the RF‐shield is to screen external RF signals from the MRI signal. The RF‐shield changes the RF‐fields produced by the transmit coil, and as previously stated, the incident electric and magnetic RF‐fields are required as input for the CSI‐EPT algorithm. Therefore, including the RF‐shield in the model that simulates these incident RF‐fields is required. The copper RF‐shield can be approximated by a perfect electrically conducting (PEC) material.

In Ref. [31], the RF‐shield was included in the Green’s tensor functions. This is an accurate way to do this; however, different MRI systems would require different Green’s tensor functions. Furthermore, the spatial invariance of the free‐space Green’s tensor function is lost, $\hat{G}\;\left(\rho \;‐\;\rho^{\prime }\right)\;\to \;\hat{G}\;\left(\rho ,\;\;\rho^{\prime }\right)$ and this results in a significant increase in computation time because the integrals in Equations (4a, 4b, 9, and 10) can no longer be computed using the fast Fourier transform, but instead have to be computed using a convolution.

To avoid a severe increase in the computation time of the CSI‐EPT algorithm, we implemented the RF‐shield for the incident fields numerically using mirror currents. At the location of the PEC, the tangential electric field should be zero. To accomplish this for the circular RF‐shield, we assume that it is infinitely long in the *z*‐direction. From there, we follow Ref. [32], where an improved placement of the mirror currents for a first‐order approximation of circular planes is given as

(11) $$d=\frac{R_{\mathrm{PEC}}^{2}}{R_{S}},$$

where *d* is the distance from the mirror source to the center of the birdcage coil, $R_{\mathrm{PEC}}$ is the radius of the RF‐shield, and $R_{S}$ is the distance from the center of the RF‐shield to the source.

## 3 METHODS

First, CSI‐EPT was used to reconstruct tissue parameters based on simulated data. Simulations allow knowledge of the ground truth EPs, thus benchmarking the accuracy of the reconstruction algorithm. Subsequently, first CSI‐EPT reconstructions were performed from MRI measurements in a phantom.

### 3.1 Simulations

**FIGURE 1:**
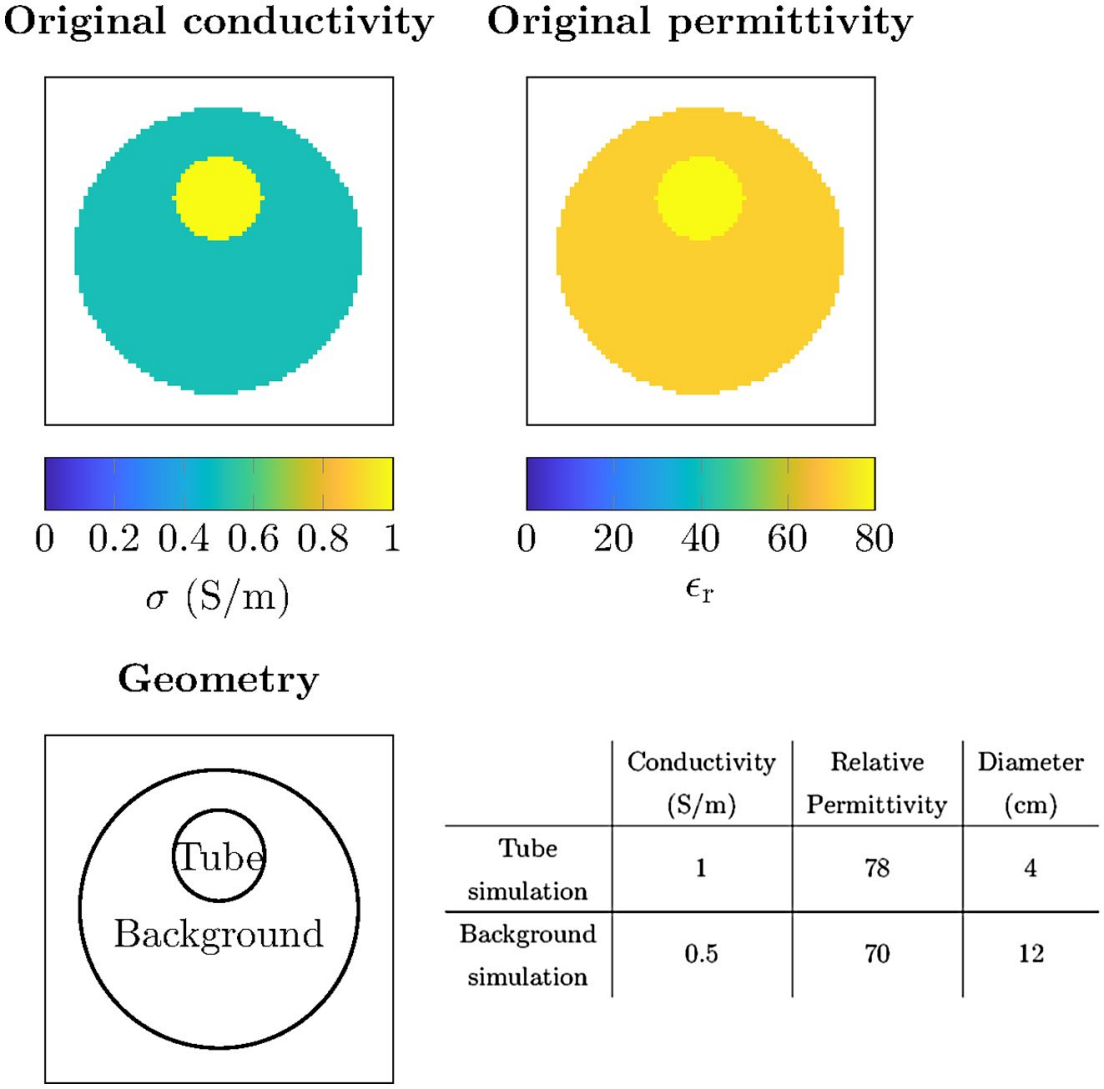
The top left shows the conductivity of the phantom and the top right shows the relative permittivity. The bottom left shows the geometry of the phantom where the inner compartment will be referred to as the tube and the other compartment is referred to as the background. On the bottom right, the table shows the conductivity and permittivity values that were used in simulations as well as the dimensions of the phantom

The performance of the modified CSI‐EPT scheme is tested first by the use of 2D line source simulations in Matlab (MathWorks, Natick, MA). The contrast that is used for these simulations is shown in Figure 1. This contrast was chosen to be asymmetrical to render the TPA invalid. With these simulated data, we investigated the following aspects:

#### 3.1.1 Impact of RF‐shield

**FIGURE 2:**
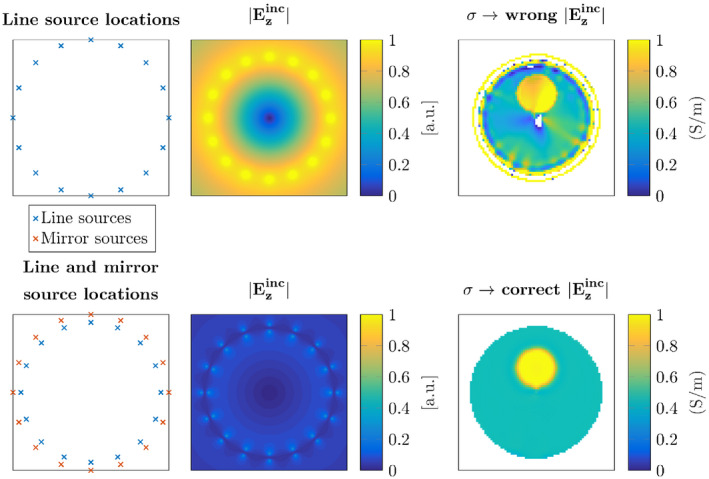
The top row shows the setup of the line sources (left) and the resulting incident electric field amplitude (middle) and the reconstructed conductivity when this incident RF‐field is used (right). The bottom row shows the same for the numerical implementation of the PEC with mirror sources. The same current is running through the line sources in both cases

The incident electric and magnetic fields were computed with and without the RF‐shield included using the setup that is shown in Figure 2. Only the quadrature mode of the birdcage coil is used for comparison between the incident field with and without the RF‐shield. The reconstructed conductivity is shown in the same figure to demonstrate the impact of using the wrong incident RF‐fields on the reconstructed electrical properties. In all the subsequent results, the RF‐shield is included in the modelling of the incident fields.

#### 3.1.2 Impact of TPC versus TPA

The effects of the transceive phase on the original CSI‐EPT algorithm, using the TPA, and the newly proposed transceive phase corrected (TPC) CSI‐EPT algorithm are investigated for 3T and 7T. In all the simulations, both the $B_{1}^{+}$ amplitude and transceive phase are separately corrupted with white Gaussian noise using realistic SNR values^33^ for the corresponding field strength (1.5T SNR = 30, 3T SNR = 54, 5T SNR = 82, 7T SNR = 119, 9T SNR = 151). We follow this approach because the $B_{1}^{+}$ amplitude and transceive phase are acquired using two different measurements each with a corresponding noise set.^34^ The noise was added to the simulation by duplicating the $B_{1}$ maps and corrupting both the real and imaginary parts separately. From one pair of corrupted real and imaginary values, the $B_{1}^{+}$ amplitude was extracted. The other pair of real and imaginary values was corrupted with a different noise set and used to construct the transceive phase. In all the simulations, total variation regularization was used during the minimization, as described in Ref. [22].

To obtain a measure of the quality of the reconstructed electrical properties, the mean absolute error is computed. This is performed within a region of the phantom as indicated by the black box in Figure 4 (see below). We define the mean absolute error as

(12) $${\mathrm{Err}}_{\sigma }=\frac{1}{N}\sum_{N}\frac{\left|\sigma_{\mathrm{true}}‐\sigma_{\mathrm{recon}}\right|}{\sigma_{\text{true}}}\cdot 100\%,$$

where ${\mathrm{Err}}_{\sigma }$ defines the mean absolute error in the conductivity, $\sigma_{\mathrm{true}}$ is the original conductivity of the phantom, $\sigma_{\mathrm{recon}}$ is the reconstructed conductivity, and *N* is the number of voxels within the region indicated by the black box.

After these 2D line source simulations, a 3D FDTD simulation package (Sim4Life, ZMT, Zurich, Switzerland) is used to investigate the effects of the 2D assumption that is used in this version of the CSI‐EPT algorithm. This assumption states that in the center plane of the transmit coil the RF‐fields have only the $E_{z},\;B_{x}$ and $B_{y}$ components. Before using measured data, we want to characterize the error that is made by using this 2D assumption.

For these simulations, the same phantom as for the 2D line source simulations was constructed, the specifications are shown in Figure 1. The simulated transmit coil is a 16‐rung high‐pass birdcage coil with a diameter of 72 cm and a rung length of 42 cm. The endrings have a width of 8 cm and the included RF‐shield has a length of 70 cm and a diameter of 74 cm. The birdcage coil is driven in quadrature mode. From these simulations, the incident RF‐fields and the $B_{1}^{+}$ magnitude and transceive phase with the phantom in the simulation are used as inputs for the CSI‐EPT algorithm.

### 3.2 Measurement

Finally, CSI‐EPT reconstructions from MRI measurements using a 3T system (Igenia, Philips, Eindhoven, Netherlands) were performed to show the potential of this method on in vivo data. Since there is no ground truth available with measurements, unlike the simulations, the CSI‐EPT reconstructions are compared to the standard Helmholtz‐based EPT method, where a 7‐point kernel is used.^18^, ^28^

For the measurements, a phantom was constructed with the same dimensions as the phantoms shown in Figure 1. The phantom was agar based and NaCl was added to give the two compartments different conductivity values. In the tube, 5.5 gr/L of NaCl was added, leading to a conductivity of 0.9 S/m at $21^{\circ }$C. For the background, 2.5 gr/L was added resulting in a conductivity of 0.41 S/m at $21^{\circ }$C.^35^

An AFI sequence was used to obtain the $B_{1}^{+}$ amplitude.^36^ The transceive phase was acquired with two spin echoes with opposite gradient polarity, this reduces the phase contribution due to the eddy currents.^28^ The body coil was used for transmission and a head coil was used for reception.

A vendor‐specific algorithm (Philips, Constant Level of Appearance‐CLEAR) was used to convert the receive phase measured with the head coil to the body coil.^37^ Using this algorithm, it is as if the body coil was used for both transmitting and receiving. The benefit is that using the head coil during reception significantly increases the SNR of the measurements. The sequence parameters that were used are noted in Table 1.

**TABLE 1:** Scanner parameters for the AFI and SE sequence

Parameter	AFI	SE
FoV	$200\;\times \;200\;{\mathrm{mm}}^{2}$	$200\;\times \;200\;{\mathrm{mm}}^{2}$
Resolution	$2.5\;\times \;2.5\;\times \;3\;{\mathrm{mm}}^{3}$	$2.5\;\times \;2.5\;\times \;2.5\;{\mathrm{mm}}^{3}$
TR1	50 ms	1000 ms
TR2	250 ms	–
TE	2.7 ms	5 ms
Flip angle	$65^{\circ }$	$90^{\circ }/180^{\circ }$

To correctly model the incident RF fields, the $B_{1}^{+}$ amplitude and the phase difference between the two ports of the birdcage coil were extracted from the log file created by the scanner.

Finally, the mean (*μ*) and standard deviation (*σ*) are given for the reconstructions of the measured data. These values are computed for the two different compartments of the phantom. Since standard Helmholtz MR‐EPT is not able to reconstruct the boundaries properly,^18^ we report the mean and standard deviation in the case the boundary between the background and the inner tube is included and when it is excluded.

## 4 RESULTS

The effect of the mirror currents is shown in Figure 2. It can be seen that the magnitude of the electric field significantly decreases for equal currents running through the line sources. Furthermore, it can be seen that the electric field goes to zero at the location of the RF‐shield. The inclusion of the RF‐shield, even with a numerical approximation, results in a more realistic model of the incident RF fields which helps with the practical implementation of the CSI‐EPT method. This is also observed in Figure 2, where the contrast is reconstructed using the incident field with and without the RF‐shield (ie, the total RF fields were calculated with the RF‐shield present).

**FIGURE 3:**
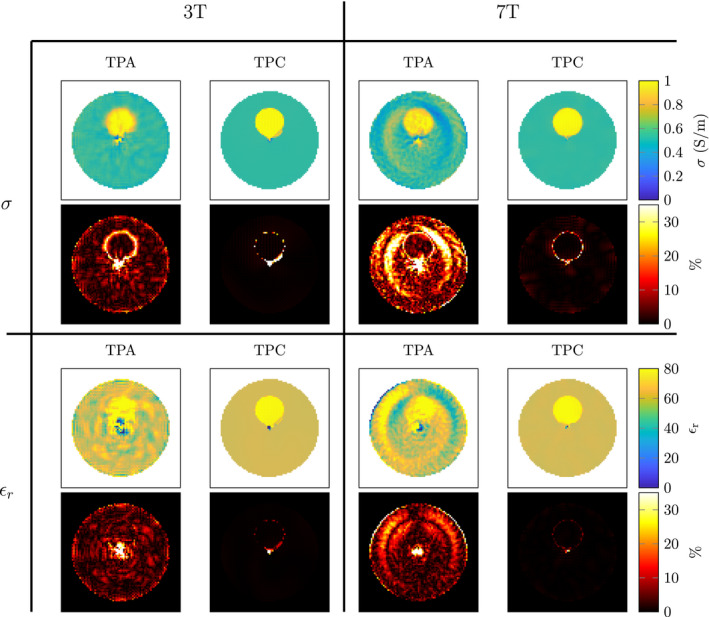
The top row shows the conductivity reconstructions with the corresponding absolute error maps below them. The reconstructions were performed with the TPA and the newly proposed method, indicated with TPC, both at 3T and 7T. The third row shows the permittivity reconstructions with the corresponding error maps below them

CSI‐EPT reconstructions from the 2D line source simulations at different field strengths are shown in Figure 3. It can be observed that the error due to the TPA increases with increasing field strength. This is because of the increasing invalidity of the TPA at higher field strengths, whereas for the transceive phase corrected CSI‐EPT algorithm this error is not present. Furthermore, the reconstruction for the transceive phase corrected CSI‐EPT algorithm improves due to the higher field strength with its inherently increased sensitivity.

**FIGURE 4:**
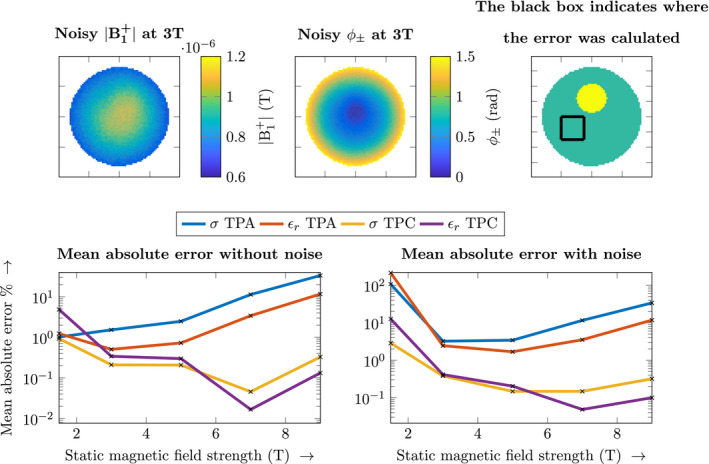
The top left shows an example of a simulated noisy $B_{1}^{+}$ amplitude map at 3T. The top middle figure shows the corresponding simulated noisy transceive phase. The top right figure shows where the mean absolute error was computed. The bottom row shows the mean absolute error in the conductivity and permittivity on a log scale vs the static magnetic field strength, where the left figure is without noise in the simulation and the right figure is with noise. The 3T and 7T reconstructions of these data points can be seen in Figure 3

This is further illustrated in Figure 4, where the effect of these two field strength dependent factors is shown. The mean absolute error of the reconstructions at different field strengths for both the standard CSI‐EPT (with TPA) scheme and the newly proposed TPC CSI‐EPT are shown. Furthermore, examples of the noise corrupted simulated $B_{1}^{+}$ magnitude and transceive phase are shown. From the line plots, we can see the impact of the noise at lower field strengths, because of the lower sensitivity, and the TPA at the higher field strengths. When the TPC CSI‐EPT algorithm is used, the higher intrinsic sensitivity at the higher field strengths can be utilized.

**FIGURE 5:**
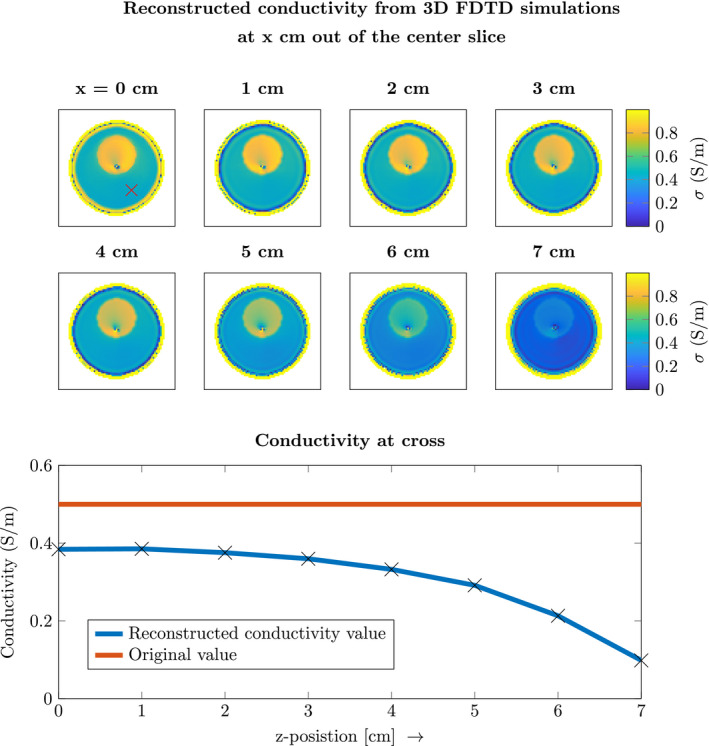
The top left figure shows the reconstruction of the conductivity in the center of the birdcage coil for the 3D FDTD simulations. The seven subsequent figures are reconstructions each 1 cm more out of the center slice of the birdcage coil. The bottom figure shows the value of the actual conductivity and the reconstructed value at the red cross marked in the top left figure

Figure 5 shows the conductivity reconstructions from the 3D FDTD simulations. This is to demonstrate the effect that 3D electromagnetic fields have on the reconstructed contrast since we are using the 2D CSI‐EPT. The top left reconstruction is taken from the center slice of the birdcage coil. The subsequent reconstructions are from slices 1 cm further out of the middle. All these reconstructions were computed separately as 2D slices and not as a 3D volume.

The bottom plot of Figure 5 shows the conductivity value across the reconstructions for the point indicated with the red cross in the top left reconstruction. From this plot, it can be seen that the conductivity value is underestimated. The underestimation increases for more peripheral locations along the cylindrical axis of the birdcage coil (ie, moving toward the endrings of the birdcage coil). This arises from the fact that the 2D EM field approximation becomes increasingly more invalid. Furthermore, the phantom is not 2D thus at the end of the phantom the RF‐field is not 2D E‐polarized.

**FIGURE 6:**
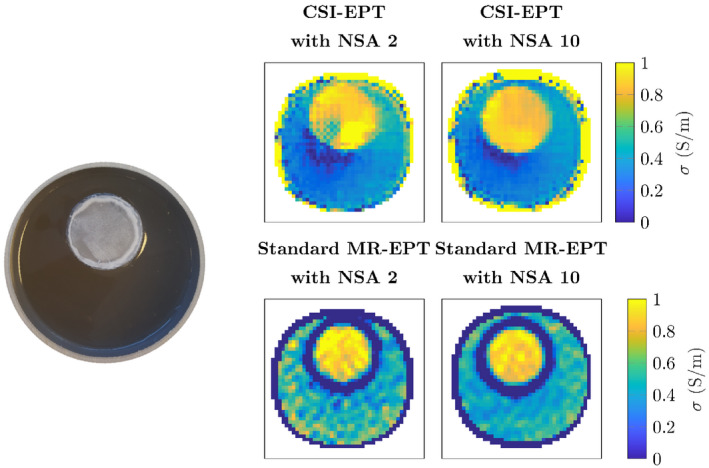
On the left is a picture of the phantom that was made for the measurements. The top left shows the conductivity reconstruction for the CSI‐EPT reconstruction with an NSA of 2, the top right shows the reconstruction for NSA = 10. The bottom two figures show the standard Helmholtz MR‐EPT reconstruction. The left shows the NSA = 2 reconstruction while the right shows the NSA = 10 reconstruction

Figure 6 shows the reconstructed conductivity maps from MRI measured data for the proposed CSI‐EPT method and the standard Helmholtz MR‐EPT as a reference. For both methods, reconstructions were performed using a number of signal averages (NSA) of 2 and 10. The comparison between the two EPT methods was performed because there is no ground truth of the contrast available, but only an average value based on the NaCl concentrations. With NSA 10, MR‐EPT shows good quality maps, while these are noisier for the NSA 2 case. This can also be observed from Table 2. Furthermore, we observe that the standard deviation of the CSI‐EPT reconstruction is always lower compared to the MR‐EPT reconstruction. Additionally, in both the NSA, 2 & 10 reconstructions of the CSI‐EPT have the same mean values, while this is not the case for the MR‐EPT reconstruction. However, the MR‐EPT reconstructs the mean value of the background better than CSI‐EPT, which underestimates the conductivity. Nonetheless, this demonstrates the feasibility of CSI‐EPT in a realistic setting using data acquired in clinically feasible scan time.

**TABLE 2:** Mean and standard deviation (*SD*) of the conductivity reconstructions from MRI data

Reconstruction used	Mean inner tube	*SD* inner tube	Mean background	*SD* background
CSI‐EPT NSA = 2	0.87	0.17	0.29	0.12
CSI‐EPT NSA = 10	0.87	0.05	0.29	0.09
MR‐EPT NSA = 2	0.83	0.22	0.42	0.16
MR‐EPT NSA = 10	0.81	0.22	0.37	0.13
MR‐EPT with boundary NSA = 2	0.6	0.51	0.29	0.7
MR‐EPT with boundary NSA = 10	0.63	0.42	0.24	0.68
Reference value	0.9	–	0.41	–

**FIGURE 7:**
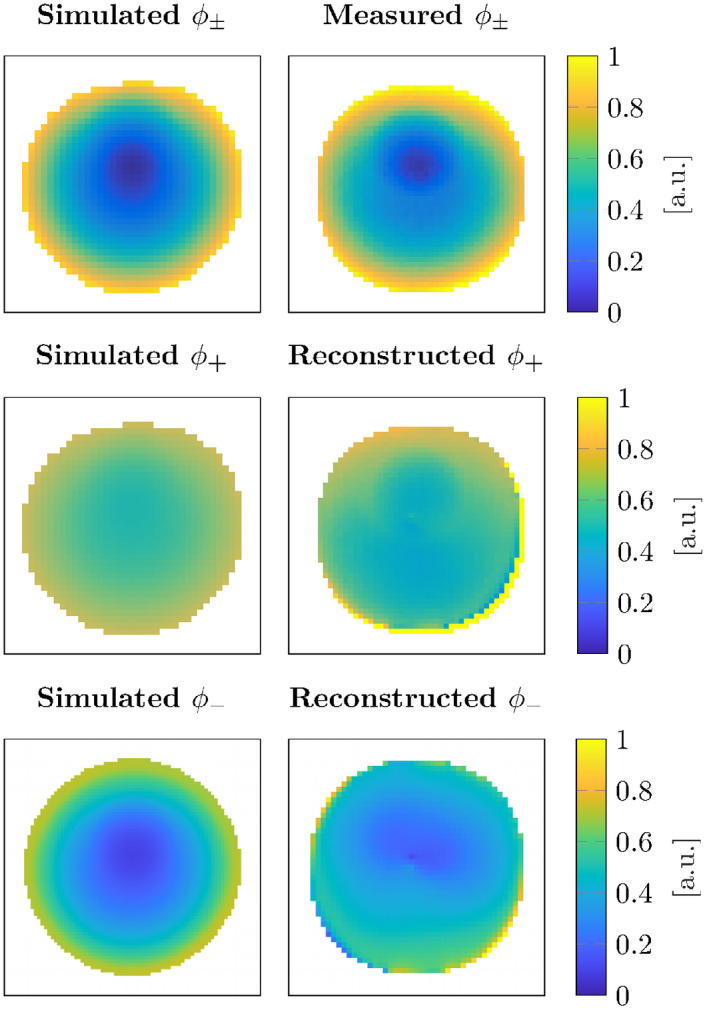
The top left shows the transceive phase from the 2D simulation. The top right shows the transceive phase measured with MRI. The middle left shows the simulated transmit phase and the middle right shows the transmit phase reconstructed by CSI‐EPT from the measurement. The bottom row shows the simulated and reconstructed receive phase

Finally, since the newly proposed method also reconstructs the receive phase during the minimization process, it is possible to compare this reconstructed phase from the measurement with the 2D line source simulated one. This together with the comparison between the 2D simulated transceive phase and the measured transceive phase is shown in Figure 7. The simulated, measured, and the reconstructed phases show good agreement. This indicates that the receive phase can be reconstructed using the TPC CSI‐EPT.

## 5 DISCUSSION

In this work, we reformulated the CSI‐EPT algorithm in terms of MRI accessible quantities, that is, the $B_{1}^{+}$ magnitude and the transceive phase. Furthermore, we showed that with this formulation we can derive the true $B_{1}^{‐}$ fields rather than the relative $B_{1}^{‐}$, which is reconstructed by using one transmit channel as a reference. Furthermore, we included a numerical implementation of the RF‐shield resulting in more realistic incident RF‐fields that are used as input for CSI‐EPT. Ultimately, with this work, we demonstrated for the first time the feasibility of CSI‐EPT conductivity reconstructions from MRI measurements at 3T. These results ultimately confirm the reduction of boundary errors and less noise compared to Helmholtz EPT as is always claimed from simulated results.

The solution to the RF‐shield was proposed to model the incident RF‐fields correctly. Another solution to implement the RF‐shield into the CSI‐EPT scheme has been proposed in Ref. [31], where instead of the free‐space Green’s tensor functions the Green’s tensor functions in the presence of a circular PEC, which represents the RF‐shield, has been used. This solution showed great improvement, however, the downside of using this method is that the computation time of the CSI‐EPT algorithm will increase drastically. Since integral approaches are already slower compared to derivative approaches, we chose to use the implementation proposed in this work, where we only include the RF‐shield in the incident RF‐fields. For the center slice of the birdcage coil, the 2D RF‐fields simulated with line sources is used and shows similar electrical properties reconstructions compared to reconstructions using FDTD simulated incident RF‐fields.

The TPA is valid at lower field strengths, as a result, we can reason that the error in the reconstruction from Figure 4 for the lower field strengths is dominated by the low SNR. For higher field strengths, the error in the standard CSI‐EPT algorithm increases because the TPA is no longer valid. In Figure 3, these effects can also be observed in the electrical properties. In the 3T reconstructions, the error is predominantly due to the low sensitivity, while at the 7T reconstruction more global over and underestimations of the conductivity and permittivity can be seen. When using the TPC CSI‐EPT, the transceive phase is no longer negatively affecting the reconstruction. Furthermore, we observe that at the lower field strengths the conductivity reconstruction has a lower error compared to the permittivity reconstruction. The displacement current directly scales with the frequency.^38^ Therefore, at higher static magnetic field strength, the imprint of the permittivity on the contrast increases, possibly allowing for higher quality permittivity reconstructions at higher field strengths.

With the standard CSI‐EPT algorithm and standard Helmholtz EPT, there was a trade‐off for the field strength to use when measuring with a standard birdcage setup. At lower field strengths, the sensitivity is poor, while at higher field strengths the TPA is not valid. With the TPC CSI‐EPT, this trade‐off is no longer present since TPC CSI‐EPT is not affected by the invalidity of the TPA. Therefore, the increased SNR and inherent sensitivity of 7T for a regular widely available standard quadrature setup can be exploited. As a limitation of this work, MRI measurements were only performed at 3T and not at 7T because the specifications of transmit coil at 7T are not available. As shown in Figure 2, an incorrectly simulated background RF‐field would lead to an incorrect contrast reconstruction. Therefore, measuring at 7T with no good knowledge of the transmit coil for the simulations would result in poor reconstructions.

In each of the eight reconstructions, in Figure 3, it can be noted that reconstruction in the center of the phantom has a larger error compared to the rest of the reconstruction. This is inherently due to the design of the birdcage coil, especially in quadrature and reverse quadrature mode. In these modes, the electric fields constructed by each separate rung of the birdcage coil destructively interfere in the center of the coil creating an electric field that has an almost zero magnitude. This local minimum in the electric field, as can be seen in Figure 2, results in a poor reconstruction in this area. To improve this, either a different antenna setup could be chosen to create a different electric field distribution or a dielectric pad could be used to move the local minimum in the electric field.^21^, ^39^

Figure 5 shows how realistic three‐dimensional (3D) electromagnetic fields affect the reconstruction. The center slice of the birdcage coil is the part that resembles a 2D transverse magnetic (TM) polarized field. Slices outside of the center have larger $E_{x}$, $E_{y}$, and $B_{z}$ components that are not taken into account in this 2D CSI‐EPT algorithm. This is the cause of the underestimation of the conductivity, as can be seen by the plot at the bottom of Figure 5, especially at the outermost slices of the phantom. At the boundary between the phantom and the air, the change in conductivity and permittivity creates 3D scattered fields, and the assumption that the field is TM polarized is no longer valid. The same results were observed for the permittivity, but is omitted here to not display the same information twice.

The phantom has a contrast that is invariant over the *z*‐direction for the length of the phantom. This was done to keep the assumption that the RF‐field is 2D valid in the center slice of the birdcage coil. In Ref. [40], the effects of reconstructing fully 3D contrasts with a 2D CSI‐EPT algorithm are shown.

Possible solutions for the underestimation of the dielectric properties are to formulate the CSI‐EPT algorithm for 3D RF fields. However, this will significantly increase the computation time of the algorithm. Currently, the reconstructions are obtained within 1 minute of computation time using an i5‐6600k processor compared to multiple hours for 3D CSI‐EPT.^41^ The increase in computation time is a result of the increased problem size, increasing both the time per iteration and the total number of iterations required.^41^ If the TPC is used in the 3D implementation of CSI‐EPT, the receive phase could be calculated only at every *n*th iteration to alleviate the additional computational effort. Further, the increase in computation time can be managed by using some form of a hybrid method, where either 2D CSI‐EPT or a deep learning approach^42^ can be used as initialization for the 3D CSI‐EPT method. Another solution could be to use a tube with a reference dielectric during scanning. Then, the results can be scaled until the correct reference value is found.

From the reconstruction of the measured data in Figure 6, it is clear that the CSI‐EPT reconstruction is more noise robust compared to the standard Helmholtz MR‐EPT. Another striking feature is that for EPT a clear boundary error is present while for CSI‐EPT this error is not present. This reconstruction of the electrical properties using MRI measured data shows the feasibility of CSI‐EPT for the first time. However, for the CSI‐EPT algorithm we observe that for the measured data the conductivity of the background is underestimated and at the outer edge of the phantom the conductivity is overestimated as was expected from the reconstructions of the 3D simulations. Therefore, to improve the reconstructions for MRI measured data similar steps as discussed in the paragraph above should be taken.

From the TPC CSI‐EPT, we can also extract the reconstructed transmit and receive phase as shown in Figure 7. This was previously not possible and only the transceive phase and the receive fields with respect to a reference channel could be measured. The actual transmit and receive phase maps could be of interest for coil design and to check the performance of built coils.

Ultimately, the presented methodology enables the use of CSI‐EPT for higher field strengths with a standard birdcage setup and as a result of the reformulation to MRI accessible quantities, it could also move to be used for in vivo EPT.

## 6 CONCLUSION

In this work, the CSI‐EPT algorithm was reformulated such that the $B_{1}^{+}$ amplitude and transceive phase can serve as input data instead of the complex $B_{1}^{+}$ field. Due to this reformulation, the transceive phase assumption, which is not valid at high field strengths, is not necessary anymore. This allows for CSI‐EPT to fully exploit the benefits of the higher static magnetic field strengths with a standard quadrature birdcage coil setup.

Finally, in this work, the first MRI acquired data CSI‐EPT reconstructions are shown and illustrate a significant improvement over the standard Helmholtz MR‐EPT reconstructions. Moreover, we observed that CSI‐EPT can reconstruct the boundaries between different dielectric properties and that it is robust with respect to realistic SNR values.
